# Supplementary material for: A Novel AKT1, ERBB2, ESR1, KRAS, PIK3CA, and TP53 NGS Assay: A Non-Invasive Tool to Monitor Resistance Mechanisms to Hormonal Therapy and CDK4/6 Inhibitors
Source: Biomedicines. 2024 Sep 26;12(10):2183. doi: 10.3390/biomedicines12102183 (PMC11505462; doi:10.3390/biomedicines12102183)
Supplement: Supplementary file 1 [file biomedicines-12-02183-s001.zip › Supplementary Figure S2_revised20240912.pdf]

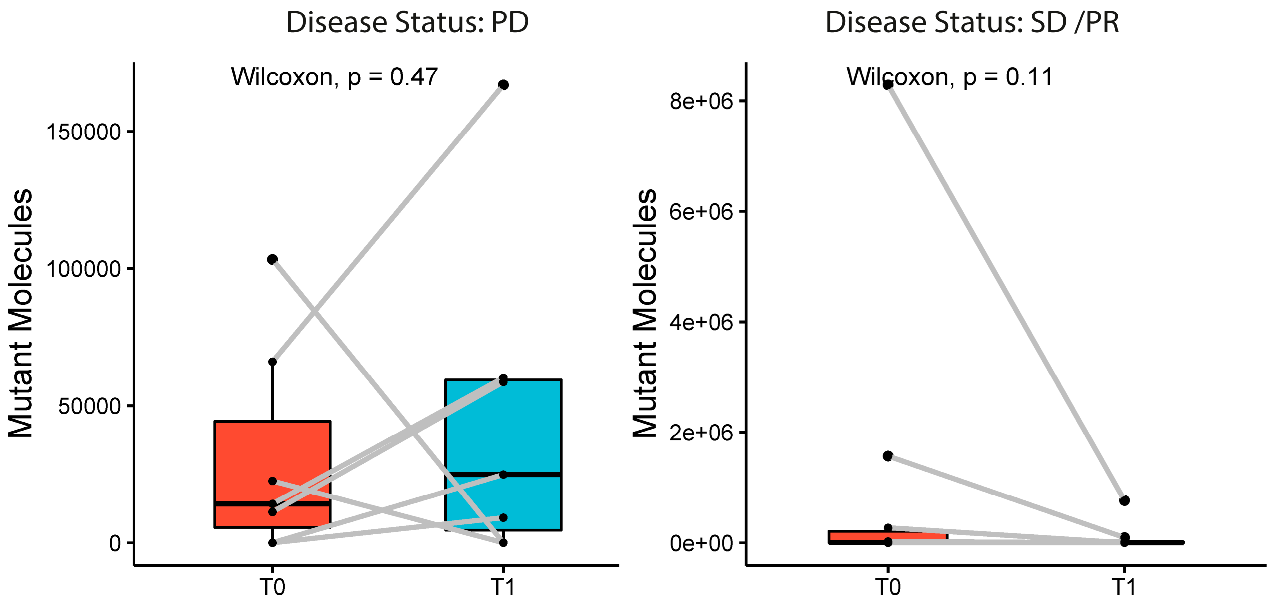

Supplementary Figure S2. The box plot shows the Wilcoxon test applied to the mutant molecules (MM) trend of alterations between T0 and T1 in patients who presented PD (left) and patients with SD or PR (right).
