# Supplementary material for: A Novel AKT1, ERBB2, ESR1, KRAS, PIK3CA, and TP53 NGS Assay: A Non-Invasive Tool to Monitor Resistance Mechanisms to Hormonal Therapy and CDK4/6 Inhibitors
Source: Biomedicines. 2024 Sep 26;12(10):2183. doi: 10.3390/biomedicines12102183 (PMC11505462; doi:10.3390/biomedicines12102183)
Supplement: Supplementary file 1 [file biomedicines-12-02183-s001.zip › Supplementary File Figure S1_QC_revised 20240906.pdf]

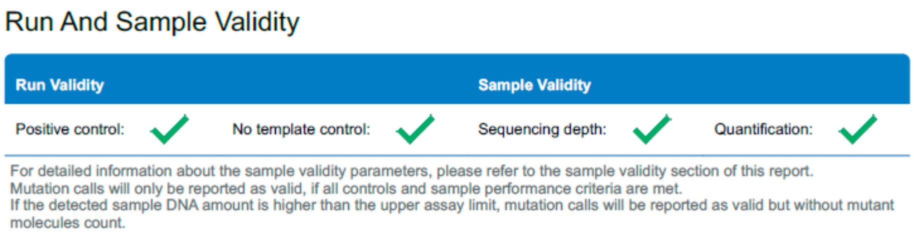

Supplementary figure S1. The figure presents a representative quality check of the Plasma-SeqSensei™ IVD Software®. The report generated by the Plasma-SeqSensei™ IVD Software® outlines four essential parameters in the quality control (QC) process. The quantification focus on the DNA input range for the PQS assay using the Breast Cancer IVD kit is between 4.3 ng and 86 ng per 116 µl, as specified by the software criteria. If a sample falls outside this linear range, the results become invalid, rendering both the mutant molecules (MM) and the quantification unreliable. Additionally, the report includes criteria for the Positive Control (PC) and No Template Control (NTC), both of which are incorporated into the library preparations from the starting plate to the sequencer. For sequencing depth, the minimum requirement is calculated as 1-fold genome equivalent (GE). Thanks to the use of Quantispike, the software can determine the genome equivalents amplified during the UID PCR process. Each genome equivalent must be covered by at least one valid UID family, which should include a minimum of four different UID family members. This coverage is essential for obtaining a valid result.
