# Supplementary material for: A Novel AKT1, ERBB2, ESR1, KRAS, PIK3CA, and TP53 NGS Assay: A Non-Invasive Tool to Monitor Resistance Mechanisms to Hormonal Therapy and CDK4/6 Inhibitors
Source: Biomedicines. 2024 Sep 26;12(10):2183. doi: 10.3390/biomedicines12102183 (PMC11505462; doi:10.3390/biomedicines12102183)
Supplement: Supplementary file 1 [file biomedicines-12-02183-s001.zip › Supplementary File Figure S3_OS_revised 20240910.pdf]

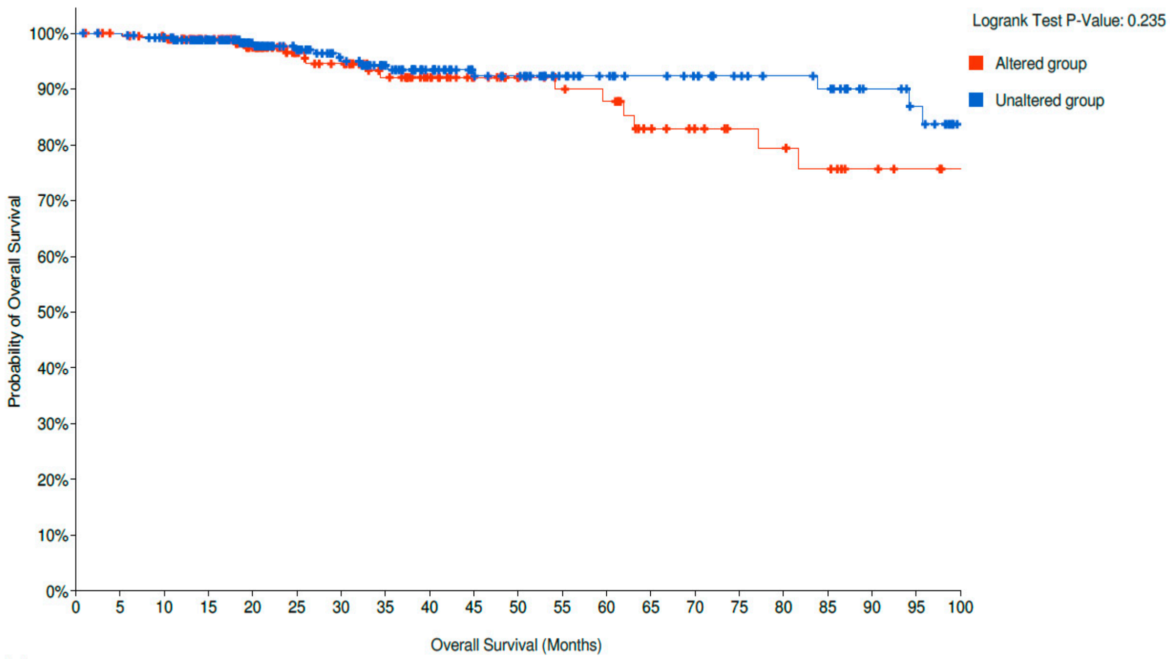

Number at risk (n)

Altered group	186	182	176	154	126	99	91	74	63	54	47	42	40	31	27	24	23	20	16	14	12
Unaltered group	249	247	236	211	175	151	134	118	99	83	79	64	58	54	51	44	41	39	31	27	16

Supplementary Figure S3. OS of *PIK3CA* mutated and WT BC patients treated with hormonal therapy (Data obtained from cBioportal PMID:29625048).
