# Supplementary material for: A Novel AKT1, ERBB2, ESR1, KRAS, PIK3CA, and TP53 NGS Assay: A Non-Invasive Tool to Monitor Resistance Mechanisms to Hormonal Therapy and CDK4/6 Inhibitors
Source: Biomedicines. 2024 Sep 26;12(10):2183. doi: 10.3390/biomedicines12102183 (PMC11505462; doi:10.3390/biomedicines12102183)
Supplement: Supplementary file 1 [file biomedicines-12-02183-s001.zip › Supplementary File Table S2_QCnext_revised 20240905.pdf]

Category	***Acceptance Range***	***Value RUN 1***	***Value RUN 2***
Sequencer ID	-/-	NB552192	
Sequencer Kit	-/-	High Output v2.5 (150-Cycle)	
Clusters Passing Filter	≥ 80%	92.5%	90.8%
Clusters Density [Clusters / mm^2]	NextSeq TM 500/550: ≤ 220k	182.0	208.0
Percentage > Q30	≥ 80%	92.7%	91.0%

Supplementary Table S2. Quality parameters of the first and the second experiment obtained by Nextseq550 instrument. The present parameters are referred to the 2 global runs including 16 samples each. The clustering passing filter is an indication of signal purity from each cluster. The cluster density is an important factor in optimizing data quality and yield. The recommended raw cluster density for NextSeq 500/550 is 170-220k/mm2 for well-balanced, base diverse libraries. The percentage >Q30 is the percentage of bases with a quality score of 30 or higher.
